# Spontaneous Intramural Duodenal Hematoma: Pancreatitis, Obstructive Jaundice, and Upper Intestinal Obstruction

**DOI:** 10.1155/2016/5321081

**Published:** 2016-11-07

**Authors:** Chalerm Eurboonyanun, Kulyada Somsap, Somchai Ruangwannasak, Anan Sripanaskul

**Affiliations:** ^1^Department of Surgery, Faculty of Medicine, Khon Kaen University, Khon Kaen 40002, Thailand; ^2^Department of Radiology, Faculty of Medicine, Khon Kaen University, Khon Kaen 40002, Thailand

## Abstract

Nontraumatic intramural duodenal hematoma can cause upper gastrointestinal tract obstruction, upper gastrointestinal hemorrhage, jaundice, and pancreatitis and may be present in patients with normal coagulation. However the pathogenesis of the condition and its relationship with acute pancreatitis remain unknown. We present a case of spontaneous intramural duodenal hematoma and a case of successful nonoperative treatments.

## 1. Introduction

Intramural duodenal hematoma was first described by McLauchlan in 1838 [1]. Intramural duodenal hematoma usually occurs secondary to blunt abdominal injury [2, 3]. Spontaneous intramural duodenal hematoma has been associated with coagulopathy, coagulating drugs, and endoscopic procedures [4–8]. However, there are many reports regarding intramural duodenal hematoma's association, with acute pancreatitis and pancreatic malignancy [9–13]. However, the association between intramural duodenal hematoma and acute pancreatitis is still unclear. In this paper, we discuss a case in which a patient presented with obstructive jaundice, upper gastrointestinal obstruction, and upper gastrointestinal hemorrhage complicated by acute pancreatitis.

## 2. Ethical Consideration

This retrospective case report was approved by the Ethics Committee for Human Research based on the declaration of Helsinki and the ICH good clinical practice guidelines. Clinical data were obtained by reviewing medical records.

## 3. Case Report

A 27-year-old Thai male was admitted to the hospital after experiencing one day of epigastric pain, hematemesis, and jaundice without evidence of previous trauma. His symptoms also resulted in upper gastrointestinal obstruction, which caused 2 kilograms of weight loss (70 to 68 kilograms) in one week. He had no history of warfarin or aspirin therapy. However, he was a heavy drinker consuming 500 mL of liquor per day.

A physical examination revealed jaundice and mild tenderness over the epigastric region. Blood tests showed a hemoglobin level of 12.3 g/dL, white blood cell count of 13,960/*μ*L, platelet count of 669,000/*μ*L, prothrombin time of 12.10 sec, INR of 1.14, activated partial thromboplastin time of 31.40 sec, lipase level of 477 U/L, and total bilirubin level of 12.1 mg/d.

**Figure 1:**
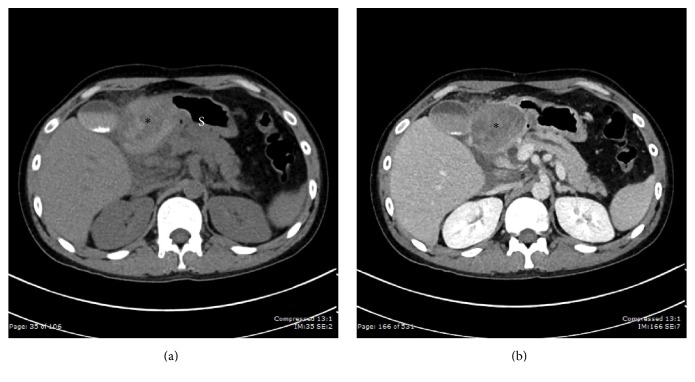
(a) Noncontrast CT scan shows a heterogeneously high-attenuation mass (*∗*) along the course of 1st, 2nd, and 3rd part of duodenum, which is compatible with duodenal hematoma. Note the surrounding fat stranding. S = stomach. (b) Contrast-enhanced CT shows the lack of enhancement within the mass (asterisk). Note the displacement of gas in gastric lumen secondary to mass.

Contrast-enhanced abdominal computed tomography revealed hematoma in the whole duodenum with duodenitis. Localized acute pancreatitis was found in the uncinate process and head of the pancreas with a small pancreatic pseudocyst causing distal common bile duct obstruction with biliary dilatation (Figures 1(a)-1(b)).

**Figure 2:**
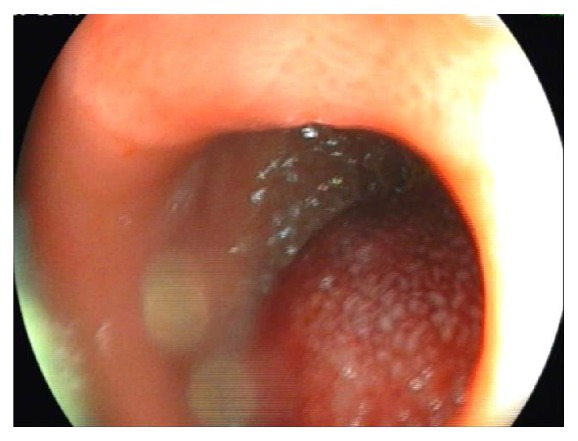
Submucosal swelling with erythematous surface extending from the duodenal bulb to the 3rd part of the duodenum causing partial duodenal obstruction: suspected intramural duodenal hematoma.

**Figure 3:**
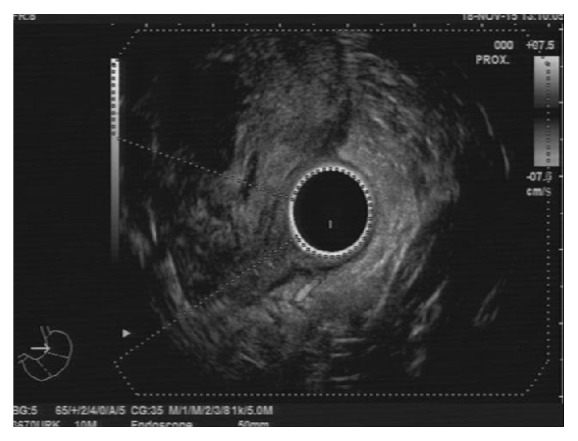
Endoscopic ultrasonography revealed avascular a heteroechoic submucosal mass at the posterolateral wall of the duodenum.

Esophagogastroduodenoscopy showed bulging, inflammation, and swelling of mucosa at the posterolateral wall of second to third part of the duodenum with partial obstruction of the duodenal lumen (Figure 2). Endoscopic ultrasonography showed a heteroechoic mass at the posterolateral wall of the duodenum (Figure 3).

The patient was diagnosed with acute pancreatitis, intramural duodenal hematoma, acute cholangitis, and upper gastrointestinal obstruction. He was conservatively treated with intravenous antibiotics and fluid replacement therapy, restricting the oral intake of food/liquids.

17 days after admission the patient was able to consume a low residual diet orally without abdominal discomfort. His jaundice had also improved. He discontinued intravenous antibiotics after 10 days of therapy. He was discharged on the 21st day after admission.

**Figure 4:**
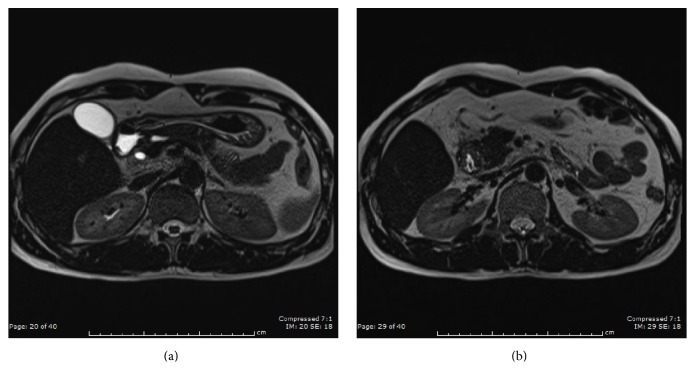
A follow-up MRI after 3 months of conservative treatment shows a nearly complete resolution of the duodenal hematoma.

Three months after admission follow-up esophagogastroduodenoscopy showed normal duodenal mucosa with a mild narrowing of the lumen at the second part of the duodenum. Magnetic resonance cholangiopancreatography revealed a distal common bile duct obstruction and revealed that the intramural duodenal hematoma, pancreatitis of head, and uncinate process had resolved (Figures 4(a)-4(b)).

## 4. Discussion

Spontaneous intramural duodenal hematoma is usually associated with coagulation abnormalities resulting from anticoagulating drugs [4–7]. If spontaneous intramural duodenal hematoma occurs in patients with normal coagulation, pancreatic diseases should be investigated [9–14].

In cases of spontaneous intramural small bowel hematoma, the rates of abdominal pain and emesis are high (84.6–100%). However, hematemesis and fever are only occasionally present (15.3–23.1%) [6]. In this case, the patient presented with abdominal pain, hematemesis, and jaundice. The only positive findings upon physical examination were jaundice and mild tenderness over the epigastric region.

CT scans, gastroduodenoscopy, and endoscopic ultrasonography revealed evidence of duodenal hematoma, pancreatitis, biliary obstruction, and a gallstone.

He was successfully treated using conservative methods. Although there has been a case report of endoscopic decompression for intramural duodenal hematoma with gastric outlet obstruction to relieve the symptoms of the obstruction [15], this procedure requires further investigation.

In this case, the disease may have developed from either alcoholic pancreatitis due to patient's history of heavy drinking or biliary pancreatitis, as there was evidence of a gallstone. It is also possible that spontaneous intramural duodenal hematoma of unknown origin caused the acute pancreatitis. But pancreatitis is likely to be the leading cause in this case, based on the patient's history of alcohol consumption and evidence of a gallstone without any coagulation abnormalities. However, it is difficult to explain the true pathophysiology.

In conclusion, the patient who presented with spontaneous intramural duodenal hematoma with acute pancreatitis with clinical jaundice, upper gastrointestinal obstruction, and upper gastrointestinal hemorrhage was able to be treated conservatively. However, the relationship between the disease and pathophysiology remains unclear.

## References

[1] McLauchlan J. (1838). False aneurysmal tumour occupying nearly the whole of the duodenum. *The Lancet*.

[2] Hayashi K., Futagawa S., Kozaki S., Hirao K., Hombo Z. (1988). Ultrasound and CT diagnosis of intramural duodenal hematoma. *Pediatric Radiology*.

[3] Jewett T. C., Caldarola V., Karp M. P., Allen J. E., Cooney D. R. (1988). Intramural hematoma of the duodenum. *Archives of Surgery*.

[4] Polat C., Dervisoglu A., Guven H. (2003). Anticoagulant-induced intramural intestinal hematoma. *American Journal of Emergency Medicine*.

[5] Tseng C.-Y., Fan J.-S., Yang S.-C. (2010). Anticoagulant-induced intramural intestinal hemorrhage. *The American Journal of Emergency Medicine*.

[6] Abbas M. A., Collins J. M., Olden K. W., Kelly K. A. (2002). Spontaneous intramural small-bowel hematoma: clinical presentation and long-term outcome. *Archives of Surgery*.

[7] Abbas M. A., Collins J. M., Olden K. W. (2002). Spontaneous intramural small-bowel hematoma: imaging findings and outcome. *American Journal of Roentgenology*.

[8] Grasshof C., Wolf A., Neuwirth F., Posovszky C. (2012). Intramural duodenal haematoma after endoscopic biopsy: case report and review of the literature. *Case Reports in Gastroenterology*.

[9] Veloso N., Amaro P., Ferreira M., Romãozinho J. M., Sofia C. (2013). Acute pancreatitis associated with a nontraumatic, intramural duodenal hematoma. *Endoscopy*.

[10] Shiozawa K., Watanabe M., Igarashi Y., Matsukiyo Y., Matsui T., Sumino Y. (2010). Acute pancreatitis secondary to intramural duodenal hematoma: case report and literature review. *World Journal of Radiology*.

[11] Silva J. D., Veloso N., Godinho R., Gonçalves L., Medeiros I., Viveiros C. (2012). Fatal acute pancreatitis following sclerosis of a bleeding duodenal ulcer complicated by an intramural duodenal hematoma. *Revista Espanola de Enfermedades Digestivas*.

[12] Chang C.-M., Huang H.-H., How C.-K. (2015). Acute pancreatitis with an intramural duodenal hematoma. *Internal Medicine*.

[13] Khurana T. (2014). Intramural duodenal hematoma with acute pancreatitis in a patient with an overt pancreatic malignancy. *ACG Case Reports Journal*.

[14] Jones W. R., Hardin W. J., Davis J. T., Hardy J. D. (1971). Intramural hematoma of the duodenum: a review of the literature and case report. *Annals of Surgery*.

[15] Lee J. Y., Chung J. S., Kim T. H. (2012). Successful endoscopic decompression for intramural duodenal hematoma with gastric outlet obstruction complicating acute pancreatitis. *Clinical Endoscopy*.

